# The Effect of Sphincter Pressure and Anal Length on Surgical Decision in Chronic Anal Fissure

**DOI:** 10.3390/jcm14113805

**Published:** 2025-05-29

**Authors:** Fadime Kutluk, Sefa Ergün, İsmail Mihmanli, Süleyman Demiryas, Rauf Hamid, Asiye Perek

**Affiliations:** 1 Department of General Surgery, Mengucek Gazi Training and Research Hospital, Erzincan 38300, Türkiye; 2 Department of General Surgery, Cerrahpasa Medical Faculty Hospital, Istanbul University-Cerrahpasa, İstanbul 34093, Türkiye; sefa.ergun@iuc.edu.tr (S.E.); suleyman.demiryas@iuc.edu.tr (S.D.); aperek@istanbul.edu.tr (A.P.); 3 Department of Radiology, Cerrahpasa Medical Faculty Hospital, Istanbul University-Cerrahpasa, İstanbul 34093, Türkiye; mihmanli@yahoo.com; 4 Radiology Department, Sungurlu State Hospital, Çorum 19300, Türkiye; drraufhamid@hotmail.com

**Keywords:** chronic anal fissure, endoanal ultrasonography, anal manometry, lateral internal sphincterotomy

## Abstract

**Background/Objectives**: Chronic anal fissure, an anorectal condition, is characterized by internal anal sphincter fibers at the base of the fissure. The lifetime prevalence is 7–8%. Medical management and lifestyle modifications are the first-line treatments, whereas chemical or surgical internal anal sphincterectomy is an option for refractory cases. The aim of this study was to evaluate the impact of surgical anal canal length and sphincter pressure on postoperative symptom relief and continence outcomes. **Methods**: A total of 26 patients diagnosed with chronic anal fissures were selected for surgical treatment. Five patients voluntarily withdrew, leaving 21 cases for analysis. In patients undergoing open lateral internal sphincterotomy, anal canal length and sphincter pressure were assessed preoperatively and three months postoperatively using endoanal ultrasonography and high-resolution anal manometry. **Results**: Postoperatively, all patients experienced complete resolution of pain and bleeding. The expected decrease in resting pressure was not significantly associated with sex. A significant weight gain was observed postoperatively (*p* = 0.001). Although a significant decrease was observed in preoperative sphincter resting pressure and VAS scores, Wexner scores remained unchanged. Preoperatively and postoperatively, mean sphincter pressure was significantly lower in the spontaneous vaginal delivery group than that in the non-spontaneous vaginal delivery group (*p* < 0.05). Patients with short anal canal lengths had greater pressures reductions, though not statistically significant. **Conclusions**: Lateral internal sphincterotomy is an effective and safe treatment for chronic anal fistula when performed by experienced surgeons. Anal canal length may influence postoperative complications and surgical decisions. Larger studies are needed to confirm these findings.

## 1. Introduction

Anal fissures can be either acute or chronic conditions that cause severe pain and bleeding, significantly impacting a patient’s quality of life [1]. The primary symptom associated with anal fissures is pain associated with defecation, which can last for several hours. The pain is usually sharp, often with a tearing sensation. Linear anorectal bleeding, often noticed after defecation and typically during wiping, may also be associated with the presence of an anal fissure [2]. The annual incidence is 1.1 per 1000, with a lifetime risk of 7–8%. This condition is more prevalent in women aged 12–24 years and in men aged 55–64 years [3]. Anal fissures are diagnosed based on symptoms and physical examination. Patients with anal fissures constitute 10–15% of general surgery outpatients visits [4].

Acute anal fissures usually heal spontaneously and completely with medical and conservative treatments (dietary changes and warm water sitz baths). Adequate hydration (at least two liters of water daily at rest) and proper fiber intake are essential components of conservative management and play a crucial role in relieving symptoms and preventing progression to chronicity. A chronic anal fissure (CAF) is diagnosed if symptoms persist after six weeks of medical treatment or show intermittent improvement with recurrence. Conversion to CAFs occurs in 40% of acute anal fissures. The rupture in CAFs is deeper than that in acute anal fissures, and internal anal sphincter fibers are observed at the base of the fissure. Hardened chronic fissures, hypertrophic anal papillae proximally, and skin tags distally constitute the classic triad of CAFs. The exact etiology remains unclear, but contributing factors include trauma from hard stools, reduced posterior anal canal blood supply, and increased internal anal canal pressure.

Surgical intervention is the preferred treatment for CAF. Lateral internal sphincterotomy (LIS), advancement flaps, and fissurectomy are commonly used, with LIS being the gold standard [5]. Many conservative and medical options are available for the treatment of CAFs, such as warm water baths, stool softening agents, steroid pharmaceuticals, diltiazem, glyceryl trinitrate, and botulinum A toxin [4,5,6,7,8,9,10,11,12,13,14].

Numerous randomized controlled trials have demonstrated the superiority of LIS over topical nitrates, calcium channel blockers, and Botox for the treatment of primary CAFs. The remission rate is 88–100% and is considered the most effective treatment for primary CAFs [2]. However, no studies have evaluated the factors influencing the decision to perform sphincterotomy. This study aims to address this gap by investigating the relationship between anal canal length and postoperative outcomes, including symptom relief, complications, patient satisfaction, and changes in resting and squeezing pressures in patients undergoing LIS for CAF. In this study, the ‘anatomical anal canal’ refers to the distance between the anal verge and the dentate line, whereas the ‘surgical anal canal’ refers to the distance between the puborectal sling and the anal verge [2,4].

## 2. Materials and Methods

This prospective study was conducted in the general surgery clinic of Istanbul University-Cerrahpaşa, Cerrahpaşa Medical Faculty Hospital between 2021 and 2022. The local ethics committee of our hospital approved this study (approval number: 18152). Patients aged >18 years who were diagnosed with CAF were included in the study. Patients with a history of perianal surgery, anorectal diseases other than anal fissures (anal fistula, hemorrhoids, etc.), fecal incontinence, loss of anatomical integrity of the anal sphincter, inflammatory bowel disease, malignancy, connective tissue disease, sexually transmitted diseases, liver and kidney failure, and those with suspected perianal abscess or microabscess formation were excluded. All participants provided written informed consent before enrollment. Patients unwilling to provide consent or those who withdrew from the study were excluded. Twenty-six eligible patients who underwent sphincterectomy for CAF were randomized into study groups. A flow-diagram is added.

All patients were examined by a single general surgeon with 30 years of experience in proctology, were diagnosed with CAF, and were operated on. Preoperative pain levels, visual analog scale (VAS) scores, and Wexner incontinence scores were measured. The structures of the internal and external sphincters and sphincter defects, if any, were evaluated with a Hitachi Eub-5500 serial endoanal ultrasonography (EAUS) system (Hitachi, Tokyo, Japan) preoperatively in all patients. Anal canal length (mm) and anal sphincter resting pressure (mmHg) were evaluated using the Medtronic Given Imaging brand ManoScan 10 + 2-channel high-resolution anal manometry (HRAM) system (Medtronic, MN, USA) according to the London criteria. Each patient was evaluated preoperatively using an Olympus 190 series flexible rectosigmoidoscope (Olympus Corporation, Tokyo, Japan).

**Figure 1:**
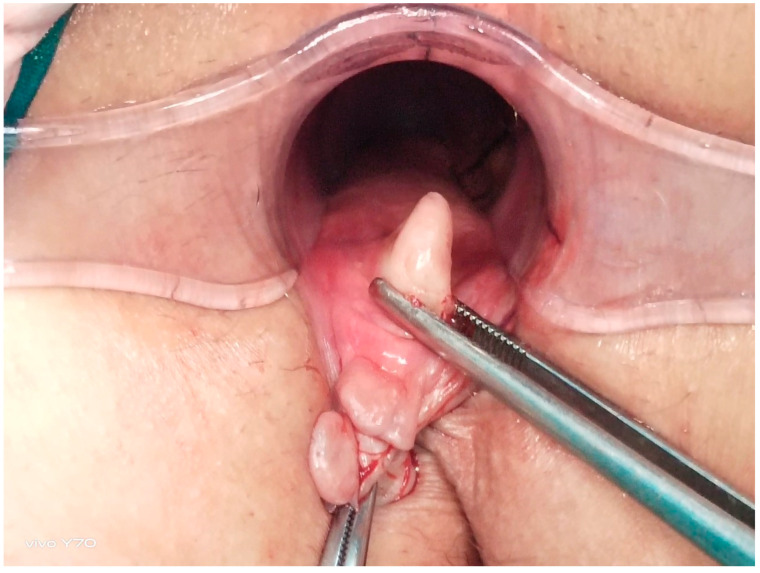
Anatomical structure of a chronic anal fissure.

**Figure 2:**
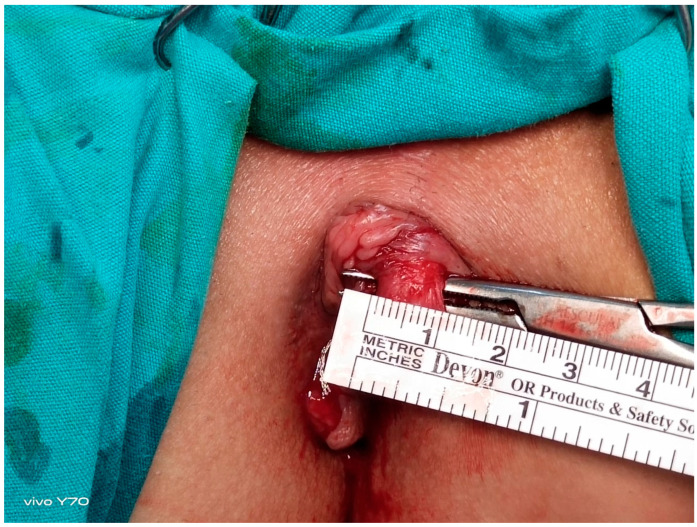
Intraoperative visualization and measurement of the internal anal sphincter muscle.

All the patients underwent preoperative bowel cleansing. An ES enema (Haver Pharmaceuticals Inc., Istanbul, Turkey) of 135 mL was administered once transanally. The same procedure was repeated before EAUS and HRAM. The operations were performed in the lithotomy position with laryngeal mask application and sevofluorane gas maintenance after intravenous administration of 1 mcg/kg fentanyl and 2.5 mg/kg propofol given at induction. Each patient underwent full-thickness left LIS up to the level of the toothed line, performed by the same surgeon using the standard open technique. The intersphincteric groove was accessed by making a 1 cm radial incision in the anoderma at the level of the LIS (3 o’clock). From this space, the internal anal sphincter was rotated laterally to medially with a dissector by moving towards the dentate line (Figure 1 and Figure 2) and was completely dissected with the help of electrocautery [6,7]. The anorectal mucosa is protected during LIS. The incision was closed using one or two absorbable sutures. Each patient received a single dose of third-generation cephalosporins preoperatively. Oral liquid food intake was initiated 6 h postoperatively, and all patients were discharged after the morning visit on the first postoperative day.

The VAS and Wexner scores were also recorded on postoperative days 1 and 10 as well as 3 months postoperatively. The sphincter structure, anal canal length, and sphincter resting pressure were reevaluated using HRAM 3 months postoperatively. The mean follow-up duration was 12 months. All patients were systematically followed up at predefined intervals: postoperative day 1, day 10, month 1, and month 3. Subsequent follow-ups were conducted every three months via telephone interviews and clinical visits, extending up to 12 months’ post-surgery. During each follow-up, continence was assessed using the Wexner score, and any symptoms or late complications (infection, bleeding, or recurrence) were documented. Throughout the 12-month follow-up period, no recurrence or new-onset incontinence was observed.

Descriptive statistics included mean, standard deviation, median, minimum and maximum values, frequency, and ratios. The distribution of variables was measured using the Kolmogorov-Smirnov and Shapiro-Wilk tests. An independent sample *t*-test was used to analyze quantitative independent data with a normal distribution. The Mann-Whitney U test was used to analyze quantitative independent data with a non-normal distribution. The paired sample *t*-test and Wilcoxon test were used to analyze dependent quantitative data. SPSS software (version 28.0) was used for the analysis. Statistical significance was set at *p* < 0.05.

## 3. Results

This prospective study initially included 26 patients with chronic anal fissure. Five withdrew during follow-up, leaving 21 patients for final analysis. All patients underwent preoperative evaluation with high-resolution anal manometry (HRAM) and endoanal ultrasonography (EAUS). HRAM was repeated three months postoperatively. The study assessed the relationship between anal canal length and changes in internal anal sphincter (IAS) resting pressure, as well as the occurrence of postoperative complications. We compared preoperative and postoperative IAS resting pressure differences between men and women, and between women with and without a history of vaginal delivery. Preoperative EAUS was used to assess sphincter anatomy, including any defects, scar tissue, or muscle thickening. HRAM was used preoperatively to measure anal canal length, IAS resting pressure, rectoanal inhibitory reflex (RAIR), and rectal compliance. Patients were between 22 and 68 years old, with a median age of 42 years. Most fissures (81%) were located posteriorly, while 9.5% were anterior, and 9.5% were both anterior and posterior. There were 13 female and 8 male patients. All had intact internal and external anal sphincters. The mean thickness of the external anal sphincter was 4.88 ± 1.19 mm. The internal anal sphincter was evaluated in three segments; upper, middle, and lower. The middle segment was the thickest, with a mean thickness of 3.39 ± 0.96 mm (Table 1 and Table 2).

Despite a significant decrease in the VAS scores of the patients postoperatively, the Jorge-Wexner Incontinence Score (JWIS) was evaluated on the first day, first month, and third month, and no significant changes were found in the pre-and postoperative scores (Table 3 and Table 4).

When the patients were evaluated pre-and postoperatively, a significant weight gain was observed after surgery compared with preoperative values (*p* < 0.05) (Table 5).

The postoperative RAIR relaxation percentage did not change significantly (*p* > 0.05) compared with preoperative values. Postoperative rectal compliance values did not change significantly (*p* > 0.05) compared to preoperative values (Table 5).

The preoperative and postoperative mean sphincter pressures did not differ significantly (*p* > 0.05) between men and women. The preoperative and postoperative lengths of the high-pressure zone (HPZ) were significantly (*p* < 0.05) lower in the group with spontaneous vaginal delivery than in the group without spontaneous vaginal delivery.

The mean sphincter pressure in the spontaneous vaginal delivery group was significantly (*p*: 0.018–0.036) lower than that in the non-spontaneous vaginal delivery group preoperatively and postoperatively (Table 6).

No significant (*p* > 0.05) difference was observed in the mean sphincter pressure at rest pre- and postoperatively between the groups with surgical anal canal ≤40 and >40 mm (Table 7).

In the group with surgical anal canals >40 mm, preoperative and postoperative RAIR relaxation percentage was significantly (*p* < 0.05) lower than the group with surgical anal canal ≤40 mm. Preoperative and postoperative HPZ length and rectal compliance did not show a significant difference (*p* 0.827–0.315) between the groups with surgical anal canal ≤40 and >40 mm (Table 7).

**Figure 3:**
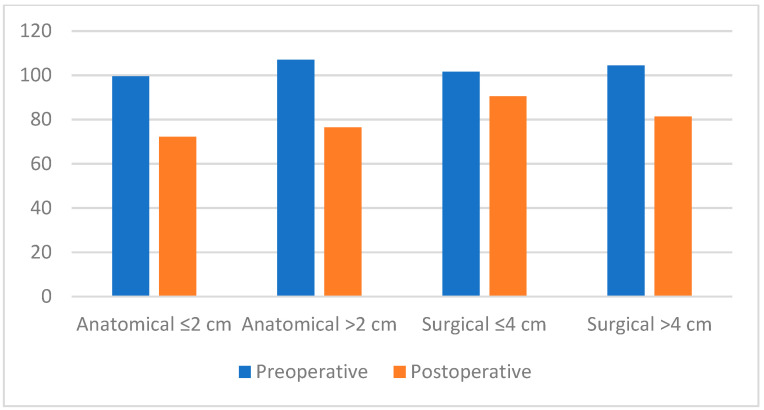
Bar chart comparing preoperative and postoperative internal anal sphincter (IAS) resting pressures according to anatomical (≤2 cm/>2 cm) and surgical (≤4 cm/>4 cm) anal canal length.

Preoperative and postoperative RAIR relaxation percentage did not differ significantly (*p* > 0.05) between groups with anatomical anal canal ≤20 and >20 mm. There was no significant difference in preoperative and postoperative minimum and maximum rectal compliance between the groups with anatomical anal canal lengths ≤20 mm and >20 mm (*p* > 0.05). Preoperative, postoperative HPZ, and rectal compliance did not differ significantly (*p* > 0.05) between the groups with anatomical anal canal ≤20 and >20 mm (Table 8, Figure 3).

## 4. Discussion

CAF is a perianal disease characterized by pain and bleeding, which significantly affects quality of life. It is a mucosal tear extending between the anal verge and toothed line. Although the exact etiology remains unclear, a widely accepted theory suggests that increased anal canal pressure and relative mucosal ischemia—caused by internal sphincter spasm following microtrauma during difficult defecation—contribute to its pathogenesis [8,9,10,11]. During the acute phase of the disease, it often heals spontaneously or completely with medical and conservative treatments (dietary changes and warm water baths). The American Society of Colon and Rectal Surgeons (ASCRS) guidelines recommend medical treatment, including stool softeners, high-fiber diet, and warm sitting baths, in the first stage of fissure treatment [12,13]. When the localization of the fissure was examined, it was observed in the posterior midline of the anal canal in 90% of cases; in 10% of cases, it was located in the anterior midline. Both anterior and posterior midline fissures were observed in 3% of cases. Chronic fissure morphology showed basal internal sphincter fibers, proximal hypertrophic anal papillae, and distal breast seizures [14,15]. LIS is considered the most effective treatment, with a 95% cure rate for CAF. In spite of the effectiveness of this technique, it has several complications, such as pain, bleeding, and abscess, which can negatively affect the quality of life of the patient.

Studies have shown the superiority of LIS over medical therapy and chemical sphincterectomy in treating CAF. However, this technique, which provides a 95% improvement, carries the risk of permanent fecal incontinence (FI) [16]. Female sex, multiple vaginal deliveries, presence of anteriorly located fissure, and history of anorectal surgery are considered high-risk factors for postoperative FI [16,17]. In our study, 13 (61.9%) patients were women, and four (30.7%) had a history of multiple spontaneous vaginal deliveries and episiotomies.

Although 7.69% (*n* = 1) and 23.07% (*n* = 3) of female patients had a history of anterior and posterior anal fissures, respectively, no complications were observed. In our study, no differences were found in the complication development rates due to sex, delivery with episiotomy or multiparous normal spontaneous vaginal delivery (NSVD), or IAS thickness values on EAUS in female patients. These findings suggest that full-thickness LIS can be performed as safely as partial LIS for incontinence.

Although CAF is frequently associated with hypertonic IAS, manometric studies have shown the presence of normotensive IAS in 20–40% of patients with CAF. Considering that the possibility of incontinence with LIS is higher in the presence of normotensive IAS, D’Orazio et al. reported in their study published in 2021 that fissurectomy and anoplasty with a V-Y advancement flap is the technique that should be used in patients with normotensive IAS. In our study, the median preoperative resting sphincter pressures of primary patients with CAF were between 105.9 mmHg and 115.6 mmHg, and we found hypertonic IAS. Therefore, we preferred LIS instead of a flap [18,19]. Brillantino et al. suggested that LIS techniques should differ by sex. In women, they recommended limiting sphincterotomy to one-fourth of the IAS length to reduce the risk of incontinence. No significant difference was observed in sphincter thickness between men and women in our study. This was confirmed by manometric evaluation. HRAM allowed detailed evaluation of sphincter function and continence, helping quantify postoperative changes. These functional results supported anatomical findings and confirmed the safety of full-thickness sphincterotomy. Therefore, all patients underwent an open full-thickness internal anal sphincterotomy limited to the distal one-third, extending up to the level of the dentate line. In our study, unlike that of Brillantino et al., a full-thickness LIS was performed, and fecal incontinence was not detected when both the Wexner and HRAM results were evaluated at the 24-month follow-up [20].

In a study by Yucel et al. on patients with anal fissures who did not heal after LIS, inadequate sphincterectomy was performed, and full-thickness sphincterectomy was advocated [21]. In our study, the resting pressures in patients with CAF were found to be significantly higher, in accordance with the literature, and a significant decrease in anal canal resting pressures was observed in the third month follow-up after LIS, as expected [22].

Endosonographic studies have demonstrated that, when the internal anal sphincter is divided into upper, middle, and lower segments, the middle segment is the thickest. In our study, the maximum localization of the internal sphincter was found to be the middle part, at 52.38%, and the average thickness in this region was found to be 3.3 mm. Therefore, we believe that the risk of inadequate sphincterectomy, which may occur in partial LIS, can be avoided when a full-thickness sphincterectomy is performed. This is supported by the fact that none of the patients in our study had an inadequate LIS when evaluated clinically and manometrically. In selected refractory cases, the use of anal dilators has been proposed as a non-invasive adjunct to sphincterotomy, with some reports suggesting favorable outcomes [23].

The anal canal was defined using two different parameters: anatomical and surgical. The anatomical anal canal is located between the anal verge and toothed line (linea dentata) and has an average length of 20 mm. The surgical anal canal is the part between the puborectal ring and anal verge, with an average length of 40 mm. Although the mean sphincter pressure at rest was 99.54 ± 23.57 mmHg in patients with an anatomical anal canal length of 20 mm or less and in patients with an anatomical anal canal length of over 20 mm, the mean sphincter pressure at rest was 107.03 ± 25.23 mmHg. We observed using postoperative control manometric measurements that the pressure was higher in patients with short anal canals than in patients with anal canal lengths over 20 mm, but it was not statistically significant (*p* > 0.05) (Table 7). In addition, the relationship of each parameter in Table 6 and Table 7 with surgical and anatomical anal canal length was examined, and we concluded that although the decrease in short surgical anal canal length was greater, it was not statistically significant. These findings highlight the potential influence of anal canal length on postoperative outcomes, which warrants further investigation. Future multicenter studies with larger sample sizes are needed to clarify whether anal canal measurements can serve as predictive markers in surgical planning for chronic anal fissure treatment. These findings highlight the potential influence of anal canal length on postoperative outcomes, which warrants further investigation. Future multicenter studies with larger sample sizes are needed to clarify whether anal canal measurements can serve as predictive markers in surgical planning for chronic anal fissure treatment. The present findings showed that the length of the anal canal and frequency of defecation did not increase postoperatively compared to the preoperative period, and this operation had no significant effect on the function of the rectum.

This study has several limitations. Firstly, the relatively small sample size limits the generalizability of the findings. To enhance the robustness of the results and statistical power, larger cohorts with more diverse populations are required. Additionally, as a single-center study, the results may not fully represent the heterogeneity observed across different clinical settings. Future multicenter studies with larger sample sizes and diverse patient populations are necessary to further explore the impact of sphincter pressure and anal length on surgical decision-making in chronic anal fissure and to validate the clinical relevance of these factors.

## 5. Conclusions

LIS is currently considered the most effective surgical treatment for CAF. Our findings confirm that full-thickness LIS can be safely performed by experienced surgeons without increasing the risk of complications, regardless of sex or history of multiparity. These findings suggest that anal canal length may contribute to postoperative risk stratification; however, its predictive utility should be validated in larger, multicenter trials. According to EAUS and HRAM evaluations, the middle segment of the IAS was the thickest, which is consistent with the literature. The combination of anatomical and functional assessments may provide a more individualized surgical approach and reduce the risk of inadequate sphincterotomy. Although the reduction in resting pressure was greater in patients with shorter anal canal lengths, this difference did not reach statistical significance. Nevertheless, this observation suggests that anal canal length may be a contributing factor in predicting postoperative outcomes and in guiding the choice of surgical technique. Further prospective, multicenter studies with larger patient populations are needed to validate these findings and clarify the clinical utility of anal canal length as a predictive marker in CAF surgery.

When comparing the lengths of the anatomical and surgical anal canals, we found that the decrease in resting pressure was greater in patients with shorter anal canals than in those with longer anal canals. However, the difference was not statistically significant. Based on this finding, we believe that a greater anal canal length may help predict postoperative complications and guide surgical technique selection to minimize risks. A prospective, randomized controlled trial with a larger sample size is needed to confirm this observation.

## Figures and Tables

**Table 1:** Parameters analyzed using endoanal ultrasonography.

Parameter	External Sphincter Thickness (mm)	Internal Sphincter Thickness (mm)			Localization Where the Internal Sphincter Is Thickest Thickness (mm)			Length Between the End of the External Sphincter and Puborectal (mm)
		Top	Middle	Bottom	Top	Middle	Bottom	
N	4.88 ± 1.19	3.13 ± 0.86	3.39 ± 0.96	3.23 ± 0.73	8	11	2	40.00 ± 7.61
%	4.9 (2.8–7)	3 (2–5.1)	3.6 (2.2–5.1)	3.2 (1.8–4.7)	(38.10)	(52.38)	(9.52)	(10.50)

**Table 2:** Intraoperative parameters.

		N	%
Fissure length (mm)		11.62 ± 2.80	12 (8–20)
Anatomical anal canal length measured from the anal verge to the dentate line (mm)		24.48 ± 4.45	25 (18–33)
Anatomical anal canal	≤2	7	(33.33)
	>2	14	(66.67)
Length from toothed line to puborectal (mm)		18.48 ± 3.33	19 (12–25)
Surgical anal canal length Measured from the anal verge to the puborectalis muscle (mm)		42.95 ± 5.22	44 (31–50)
Surgical anal canal	≤4	8	(38.10)
	>4	13	(61.90)
Distance from the intersphincteric groove to the dentate line (mm)		17.57 ± 4.31	18 (10–24)
o’clock position	6 o’clock position	17	(80.95)
	12 o’clock position	2	(9.52)
	6 + 12 o’clock position	2	(9.52)

**Table 3:** Jorge-Wexner Incontinence Score (JWIS) review.

		Preoperative		Postoperative Day 1		Postoperative Month 1		Postoperative Month 3	
		N	%	N	%	N	%	N	%
JWIS	0	21	(100.00)	19	(90.48)	21	(100.00)	21	(100.00)
	1			2	(9.52)				

**Table 4:** Visual analogue scale (VAS) analysis.

		Preoperative	Postoperative Day 1	Postoperative Day 10	Postoperative Month 1
VAS	Mean ± s.s.	7.57 ± 0.93	5.48 ± 0.60	1.95 ± 1.12	0.14 ± 0.36
	Median (Min–Max)	7 (6–10)	6 (4–6)	2 (0–3)	0 (0–1)

**Table 5:** Preoperative and postoperative manometric evaluation.

	Preop		Postop		*p*	
	Mean ± s.s.	Median	Mean ± s.s.	Median		
**Weight**	73.9 ± 19.2	72.0	78.1 ± 20.8	75.0	**0.000**	^W^
Length to HPZ	3.3 ± 1.1	3.5	3.2 ± 0.9	3.3	0.415	^E^
Verge-Center (cm)	1.4 ± 0.7	1.2	1.5 ± 0.9	1.3	0.468	^E^
**Sphincter Pressure at Rest (mmHg) Rectal Reflex**						
Average	103.2 ± 23.5	105.9	85.5 ± 28.1	82.5	**0.014**	^E^
Maximum	117.2 ± 37.0	115.6	92.2 ± 28.4	91.7	**0.017**	^E^
**Sphincter Pressure (mmHg)** Absolute Reflex						
Average	86.3 ± 15.7	89.6	66.5 ± 15.0	67.0	**0.000**	^W^
Maximum	146.6 ± 201.8	106.7	73.4 ± 17.3	74.9	**0.000**	^W^
**Defecation Attempt**						
Waste Anal Pressure (mmHg) Absolute Pressure	74.3 ± 30.5	76.9	67.7 ± 27.6	65.0	0.084	^W^
Anal Relaxation %	27.8 ± 25.7	27.0	17.5 ± 28.6	19.5	0.091	^W^
Intrarectal Pressure (mmHg)	36.4 ± 34.5	28.2	41.4 ± 28.7	38.5	0.616	^W^
RAIR Relaxation %	37.6 ± 11.9	35.0	40.7 ± 13.0	39.0	0.325	^E^
Rectal Compliance (cc/mmHg)	3.2 ± 8.8	1.6	1.5 ± 0.6	1.3	0.157	^W^

^E^ Paired sample *t*-test; ^w^ Wilcoxon test; HPZ, high-pressure zone; RAIR, rectoanal inhibitory reflex.

**Table 6:** Comparison of manometric parameters according to spontaneous vaginal delivery history.

	Spontaneous Vaginal Birth (−)				Spontaneous Vaginal Birth (+)				*p*	
	Mean ± s.s.			Median	Mean ± s.s.			Median		
**Weight**										
Preoperative	71.2	±	9.6	71.5	94.3	±	23.9	97.5	0.146	^t^
Postoperative	73.0	±	11.5	72.0	99.0	±	25.4	99.5	0.078	^t^
**Length to HPZ**										
Preoperative	4.20	±	0.67	4.20	2.85	±	0.51	2.75	**0.009**	^t^
Postoperative	3.84	±	0.59	3.70	2.43	±	0.94	2.25	**0.028**	^t^
**Verge-Center (cm)**										
Preoperative	1.42	±	0.34	1.55	1.08	±	0.43	1.05	0.194	^t^
Postoperative	1.66	±	0.30	1.70	0.75	±	0.26	0.80	**0.002**	^t^
**Mean Sphincter Pressure at Rest Sphincter Pressure Rectal Ref.**										
Preoperative	117.5	±	12.2	120.3	84.7	±	23.2	89.0	**0.018**	^t^
Postoperative	105.7	±	19.6	115.3	79.1	±	5.2	80.6	**0.036**	^t^
**Defecation Attempt Waste Anal Pressure**										
Preoperative	71.3	±	29.9	76.4	87.6	±	42.9	98.8	0.201	^m^
Postoperative	60.4	±	41.7	51.8	73.3	±	32.4	82.8	0.712	^m^
**Defecation Attempt % Anal Relaxation**										
Preoperative	30.3	±	29.3	27.0	11.8	±	22.5	2.5	0.285	^m^
Postoperative	40.1	±	22.3	38.4	9.8	±	29.2	3.5	0.142	^m^
**RAIR Relaxation %**										
Preoperative	35.8	±	4.1	35.0	25.5	±	10.8	25.0	0.130	^m^
Postoperative	37.8	±	8.4	32.0	27.8	±	7.9	25.5	0.085	^m^
**Rectal Compliance (cc/mmHg)**										
Preoperative	3.15	±	1.24	2.93	1.24	±	0.31	1.36	**0.012**	^t^
Postoperative	1.78	±	0.55	1.84	1.66	±	0.96	1.26	0.817	^t^

^t^ Independent sample *t*-test; ^m^ Mann-Whitney U test; HPZ, high-pressure zone; RAIR, rectoanal inhibitory reflex.

**Table 7:** Comparison of manometric results by surgical anal canal length.

	Surgical Anal Canal ≤40 (*n* = 9)		Surgical Anal Canal >40 (*n* = 14)		*p*	
	Mean ± s.s.	Median	Mean ± s.s.	Median		
**Weight**						
Preoperative	69.2 ± 17.4	72.0	76.9 ± 20.4	72.5	0.361	
Postoperative	71.9 ± 18.0	72.0	82.8 ± 22.3	75.5	0.243	
**Length to HPZ**						
Preoperative	3.41 ± 1.11	3.30	3.31 ± 1.09	3.60	0.827	
Postoperative	3.39 ± 0.87	3.60	2.99 ± 0.85	3.10	0.315	
**Verge-Center(cm)**						
Preoperative	1.48 ± 1.00	1.20	1.33 ± 0.36	1.37	0.617	
Postoperative	1.52 ± 1.10	1.20	1.50 ± 0.64	1.40	0.970	
**Mean Sphincter Pressure at Rest Sphincter Pressure**						
Preoperative	101.5 ± 23.3	95.2	104.4 ± 24.4	106.5	0.778	
Postoperative	90.5 ± 33.0	85.4	81.3 ± 24.2	81.4	0.483	
**Defecation Attempt Waste Anal Pressure**						
Preoperative	79.8 ± 18.1	76.9	70.8 ± 36.5	76.3	0.505	^t^
Postoperative	75.0 ± 22.7	81.7	61.8 ± 30.8	60.9	0.297	^t^
**Defecation Attempt % Anal Relaxation**						
Preoperative	20.2 ± 12.0	19.0	32.6 ± 31.0	27.0	0.517	^t^
Postoperative	12.8 ± 18.4	13.0	21.4 ± 35.3	22.0	0.585	^t^
**RAIR Relaxation %**						
Preoperative	44.3 ± 10.3	43.0	33.3 ± 11.1	34.0	0.011	^m^
Postoperative	48.0 ± 10.5	48.0	34.1 ± 11.6	31.5	0.014	^m^
**Rectal Compliance (cc/mmHg)**						
Preoperative	0.99 ± 3.50	1.56	4.54 ± 10.93	1.92	0.682	^m^
Postoperative	1.39 ± 0.44	1.30	1.63 ± 0.69	1.50	0.624	^m^

^t^ Independent sample *t*-test; ^m^ Mann-Whitney U test.

**Table 8:** Comparison of manometric results by anatomical anal canal length.

Parameter	Anatomical Anal Canal				*p*
	≤2 cm		>2 cm		
	Mean ± s.s.	Median (Min–Max)	Mean ± s.s.	Median (Min–Max)	
**Weight**	64.29 ± 15.61	72 (42–85)	81.5 ± 21.17	78 (52–117)	0.199
	66.43 ± 15.69	70 (40–87)	85.5 ± 22.48	82 (55–127)	
**Length to HPZ**	3.04 ± 0.91	2.8 (2.1–4.3)	3.66 ± 0.86	3.75 (2.2–5.2)	0.163
	3.19 ± 0.78	3.6 (1.8–3.9)	3.31 ± 0.89	3.35 (1.5–4.8)	
**Verge–center (cm)**	1.53 ± 1.12	1.2 (0.4–3.9)	1.28 ± 0.41	1.2 (0.6–1.8)	0.432
	1.44 ± 0.88	1.2 (0.6–3.1)	1.43 ± 0.64	1.4 (0.4–2.6)	
**Mean Sphincter at Rest** **Pressure (mmHg)**	99.54 ± 23.57	105.9 (73.5–134.4)	107.03 ± 25.23	110.45 (54.3–152.1)	0.824
	72.21 ± 29.66	65.4 (36.4–128.4)	76.47 ± 24.37	72.5 (44.7–127.6)	
**Attempted Defecation % Anal Relaxation**	18.71 ± 12.45	19 (−3–36)	29.17 ± 38.11	22.5 (−28–102)	0.743
	10.43 ± 20.57	13 (−24–40)	16.84 ± 32.22	17.5 (−55–61)	
**Attempted Defecation** **Intrathecal Pressure (mmHg)**	16.23 ± 20.69	22.5 (−12.6–39.1)	50.22 ± 38.49	61.2 (−6.7–109.5)	0.535
	23.81 ± 18.25	23.5 (−0.25–42.8)	51.68 ± 29.53	54.55 (−3.8–109)	
**Rectoanal Pressure Difference (mmHg)**	−60.7 ± 27.19	−48.3 (−113–35.9)	−27.03 ± 55.25	−26.2 (−106.3–93.4)	0.392
	−37.3 ± 19.66	−40.7 (−72.9–15.6)	−21.25 ± 24.44	−22.5 (−69–19.8)	
**RAIR Relaxation (%)**	45.14 ± 11.07	43 (33–60)	33.17 ± 19.28	35 (3–80)	0.242
	37.29 ± 19.18	39 (6–67)	34.75 ± 15.21	30 (17–65)	
**Rectal Compliance (cc/mmHg)**	0.81 ± 4.02	2.28 (−8.16–3.15)	5.54 ± 11.86	1.8 (−11.53–38.85)	0.708
	1.5 ± 0.30	1.45 (1.08–1.84)	4.14 ± 6.55	1.46 (0.91–24)	

HPZ, high-pressure zone; RAIR, rectoanal inhibitory reflex.

## Data Availability

Data are unavailable due to privacy or ethical restrictions.

## Abbreviations

The following abbreviations are used in this manuscript:

CAF	chronic anal fissure
LIS	lateral internal sphincterotomy
VAS	visual analog scale
EAUS	endoanal ultrasonography
HRAM	high-resolution anal manometry
RAIR	rectoanal inhibitory reflex
JWIS	Jorge-Wexner Incontinence Score
HPZ	high-pressure zone
ASCRS	The American Society of Colon and Rectal Surgeons
FI	fecal incontinence
NSVD	normal spontaneous vaginal delivery
